# Effects of enzyme + bacteria treatment on growth performance, rumen bacterial diversity, KEGG pathways, and the CAZy spectrum of Tan sheep

**DOI:** 10.1080/21655979.2020.1837459

**Published:** 2020-10-26

**Authors:** Biwei Jiang, Tian Wang, Yuxiang Zhou, Fei Li

**Affiliations:** a Agricultural College of Ningxia University, Yinchuan, China; b Ningxia Vocational and Technical College, Yinchuan, China

**Keywords:** Cellulase, compound probiotics, Tan sheep, rumen bacteria, growth performance, metagenome

## Abstract

In this study, the effects of enzyme +bacteria treatment of buckwheat straw and alfalfa on growth performance and rumen bacterial diversity was investigated, 20 three-month-old Ningxia Tan sheep with similar body weights were selected and randomly divided into two groups, 10 sheep in each group. The control group was fed with basal diet + untreated buckwheat straw and alfalfa (the ratio of buckwheat to alfalfa was 2:8), and the experimental group was fed with basic diet + cellulase (enzyme activity ≥ 10,000 U/g) + compound probiotics (enzyme to bacteria ratio 8:20). 1) The total weight gain and average daily gain of Tan sheep in the experimental group were extremely significantly higher than those in the control group (*P* < 0.01). 2). The proportion of *Firmicutes* in the experimental group was significantly higher than that in the control group (*P* < 0.05). 3). In the KEGG pathway B level, 15 genes were significantly higher than in the control group (*P* < 0.05). 4). In the CAZy level B, 12 genes were upregulated in the experimental group compared with the control group (*P* < 0.05),3 genes were downregulated (*P* < 0.05).Feeding Tan sheep with buckwheat straw and alfalfa treated with enzyme and bacteria can improve the weight gain effect, change the rumen bacterial diversity, and increase the some functional genes in the rumen. The conditions of this experiment would be beneficial to the healthy breeding of Tan sheep, and thus the methods can be used in commercial production.

## Graphical abstract

**Figure:**
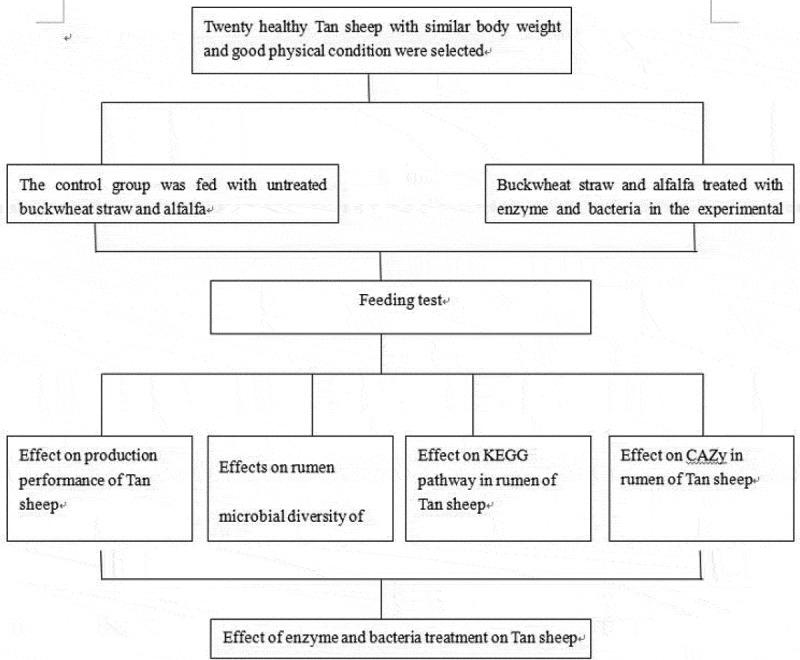

## 1. Introduction

Cellulase is composed of glucose endonuclease, glucose, and exonuclease and β-glucosidase. Together, these enzymes degrade cellulose to glucose [1]. At present, the development and application of feed enzyme preparation are the most successful examples of biotechnology in the animal nutrition and feed industry [2]. Adding cellulase to feed can effectively break plant cell wall [3], and improve the straw degradation rate [4]and nutrient composition [5,6]thereby improving ruminant growth performance, rumen fermentation type, and slaughter performance [7–10]. Buckwheat is a dicotyledonous plant of in the family *Polygonaceae* [11]. Buckwheat is a common straw crop in the southern and central arid areas of Ningxia. It not only has high nutritional and medicinal value [12], but also contains large amounts of natural inositol, flavonoids, and other active ingredients that can promote animal growth [13]. Probiotics refers to active microorganisms beneficial to the host [14].

The appropriate amount of probiotics can not only inhibit the proliferation of pathogenic microorganisms in the host intestinal tract but also maintain the balance of intestinal flora, so as to improve the production performance and improve the immune function domestic of animals. Compound probiotics can adapt to the complexity of the rumen and make up for the limitations of single microbial agents, and thus are more suitable for ruminants. The combination of *Saccharomyces cerevisiae, Bacillus subtilis*, and *lactic acid bacteria* has a synergistic effect [15]. Ding H showed that [16] added compound probiotics composed of Bacillus subtilis, photosynthetic bacteria, yeast, Bifidobacterium and Aspergillus niger in the diet of Duhan crossbred sheep, which significantly increased the acetic acid concentration in rumen fluid and the relative abundance of Bacteroides, verrucous microflora and mutrophic bacteria, and significantly reduced the relative abundance of Proteus. Wang Z C showed that [17] fed piglets with compound probiotics composed of Lactobacillus plantarum and Bacillus subtilis, which significantly increased the proliferation of beneficial bifidobacteria and Lactobacillus in the intestinal tract of piglets, and significantly inhibited the proliferation of Escherichia coli. Cellulase has been widely used in fermented roughage, but probiotic fermentation is developing rapidly.

In this experiment, cellulase and compound probiotics (*Lactic acid bacteria, Yeast*, and *Bacillus subtilis*) were used to treat two kinds of roughage (buckwheat straw and alfalfa) commonly used in the Ningxia area. The method has not previously been examined. Referring to the experimental results of Wang Meng [18], this experiment set the ratio of concentrate to Roughage at 30:70, and the best combination of buckwheat straw and alfalfa (20:80) as roughage. The effects of enzyme + bacteria treatment of buckwheat straw and alfalfa on growth performance and rumen bacterial diversity were measured, and KEGG pathways and CAZy spectrum were studied to develop and utilize straw feed resources in the husbandry of Tan sheep. The results provide a theoretical basis for commercial application.

## 2. Materials and methods

### 2.1. Fermentation of buckwheat straw and alfalfa

Proper amounts of buckwheat straw and alfalfa were prepared, crushed to 3–5 cm with a grinder, and the cellulase (enzyme activity ≥ 10,000 U/g, straw amount of 0.1%), composite probiotics (2 kg/T) and wheat bran (straw amount 1%) were accurately weighed according to the proportions in the preparation, and then with appropriate amount of water was added and evenly mixed. At the same time, the buckwheat straw and alfalfa were stirred while spraying, so that the water solution was fully mixed, and the moisture content was modulated approximately 70%. Finally, a silage wrapping machine was used to wrap feed, and the fermentation continued for 30 days.

### 2.2. Animals and feeding

The experiment was conducted at the Helanshan Cattle and Sheep Industry Group Co., Ltd. of Ningxia Agricultural Reclamation from July 2019 to September 2019. Twenty 3-month-old Ningxia Tan sheep with similar body weights and in good health were selected and divided into two groups, with 10 sheep in each group. The ratio of concentrate to roughage was 3:7. The control group was fed with basic diet + untreated buckwheat straw and alfalfa (the ratio of buckwheat to alfalfa was 2:8). The experimental group was fed with basic diet + cellulase (enzyme activity ≥ 10,000 U/g) + buckwheat straw and alfalfa treated with composite probiotics (the enzyme to bacteria ratio was 8:20). The pre-feeding period was 15 days, and the normal feeding period was 60 days. On the 60^th^ day of the formal period of the experiment, rumen fluid was collected from the mouths of Tan sheep for the determination of rumen *Lactobacillus*. The experimental diet was formulated according to the feeding standard and production practices for mutton sheep in the agricultural industry standards (NY/T 816–2004). The composition and nutrition levels of the diets are shown in Table 1. The ratio of concentrate to roughage was 30:70, and the ratio of buckwheat straw to alfalfa was 20:80. Before the experiment, the sheep were treated with insecticide and other routine epidemic prevention measures, and the housing was disinfected and cleaned regularly. The pre-feeding period was 15 days; the normal feeding period was 60 days, and the sheep were fed twice daily in the morning and evening (06:30 and 18:00).Drinking water was available ad libitum.

**Table 1.** Composition and nutrient levels of diets (DM basis)%

Raw material	Diet composition	
	Control group	Trial group
Alfalfa	56	56
Buckwheat straw	14	14
Corn	15	15
Soybean meal	10	10
Wheat bran	1	1
Jute cake	2	2
NaCl	1	1
Premix^1)^	1	1
Total	100	100
Nutrient levels^2)2^		
ME MJ/kg	9.39	9.83
CP	11.81	12.82
Ca	0.92	1.32
P	0.24	0.48

^1)^Each kg of premix contains: va 200,000–400,000 iu, ve 500,000–2,000,000 iu, vd330000-80,000 iu, copper 400–600 mg, manganese 800–1600 mg, zinc 1200–2400 mg, iodine 6–80 mg, cobalt 5–40 mg, selenium 10–25 mg, calcium 20–300 g, phosphorus 20–150 g, sodium chloride ≥ 15%, water ≤ 10%.

^2)^ME was a calculated value,while the others are measured values.

### 2.3. Sample collection

From the beginning of the feeding period, the given and residual feed amounts were recorded every day, and the dry matter intake was calculated. On the mornings of the beginning and ending days of the experiment, the weights of the test sheep on an empty stomach were recorded as the initial weight and the ending weight. The average daily gain (ADG) and feed weight ratio (F/g) of each sheep were calculated. The rumen fluid was collected on an empty stomach in the morning of the ending day of the feeding experiment. To prevent saliva contamination, the former portion was discarded, and then approximately 100 ml of rumen fluid was collected. Each rumen fluid sample was filtered through four layers of thin gauze and stored in liquid nitrogen. After the feeding test, the sample sent to Guangzhou Jidio Biotechnology Co., Ltd. for Illumina HiSeq platform sequencing.

### 2.3. Calculations and statistics

After the data for growth performance, bacterial diversity and abundance were recorded in Excel, SAS 8.2 software was used for analysis of variance, followed by LSD method tests for multiple comparisons; *P* < 0.05 was used as the standard for significance. Metagenomic data were compared with unigenes through diamond software (threshold value ≤ 1e-5). Gene abundance tables were collected to calculate the abundance information from comparison results using different databases to analyze and compare the functional differences among groups.

## 3. Results and discussion

### 3.1. Effects of enzyme + bacteria treatment on growth performance of Tan sheep

Table 2 shows that compared with the initial body weight, the experimental group and the control group had increased final body weights, but there was no significant difference (*P* > 0.05). The total weight gain, average daily gain and feed weight ratio of the experimental group were significantly different from those of the control group (*P* < 0.01). The total weight gain of the experimental group was 48.16% higher than that of the control group; the average daily gain was 44.44% higher than that of the control group, and the feed/weight ratio was decreased by 33.97%. The results suggest that the feeding of buckwheat straw and alfalfa treated with enzymes and bacteria improved the weight gain of Tan sheep. The growth performance of ruminants can be improved by using cellulase to treat roughage. Studies have shown that feeding animals with straw treated with cellulase can improve the average daily gain and dry matter intake and can reduce the feed-to-weight ratio [19].

**Table 2.** Effect of compound bacteria treatment of buckwheat straw and alfalfa on growth performance of Tan sheep

Items	Control group	Trial group
Initial body weight (kg)	33.34 ± 1.85	32.12 ± 1.65
Final body weight (kg)	38.48 ± 2.58	39.76 ± 1.36
Total gain (kg)	5.15 ± 1.23^A^	7.63 ± 1.43^B^
ADG (g/d)	90.12 ± 0.02^A^	130.25 ± 0.03^B^
DMI (g/d)	665.09 ± 0.02	634.32 ± 0.02
F/G	7.38 ± 3.50^A^	4.87 ± 1.89^B^

The same letter or no letter in shoulder mark of peer data shows no significant difference (*P* > 0.05), different letters show significant difference (*P* < 0.05), and different capital letters show significant difference (*P* < 0.01).The same as blow

Compared with using cellulase alone, adding compound probiotics in the treatment process can give the feed an aromatic flavor, thereby increasing the palatability of the feed and significantly increasing the feed intake of the animals [20]. Studies have shown that probiotic fermented feed has significant effects on lamb supplementary feeding and adult sheep fattening [21]. In a previous report where researchers fed pea feed fermented by *Lactobacillus* brucelli 11a44 to lambs, the daily weight gain of the lambs was 84 g, significantly higher than that of the control group, and the production performance was significantly improved [22], consistent with the results of this experiment. In the present study, buckwheat straw and alfalfa were treated by enzyme + bacteria fermentation, and the growth performance indexes of weight gain by Tan sheep were significantly improved. The results showed that enzyme + bacteria fermentation treatment of buckwheat straw and alfalfa can improve the utilization rate of straw, thus improving the growth performance of Tan sheep.

### 3.2. Effects of enzyme + bacteria treatment on rumen bacterial diversity of Tan sheep

**Figure 1.**
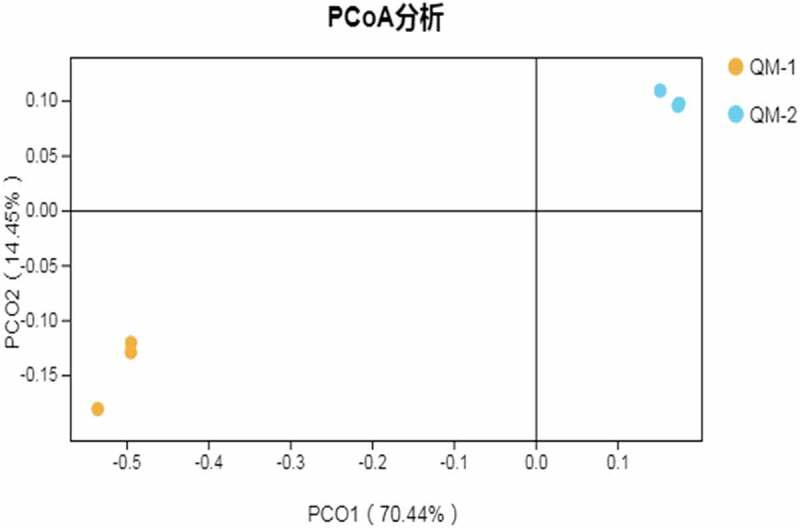
Principal coordinate analysis of OTUs of rumen bacteria

Note: QM-1 is the control group and QM-2 is the experimental group.

Table 3 shows that the coverage of the control group and the experimental group was greater than 0.98; this accurately reflects the bacterial composition in the rumen of Tan sheep. The number of species and the total number of OTUs in the experimental group were 9.00% and 3.77% lower than those in the control group, but the difference was not significant (*P* > 0.05). The results showed that feeding Tan sheep with buckwheat straw and alfalfa after enzyme + bacteria fermentation did not affect the number of rumen bacteria species or OTUs. According to Table 4, the Chao index and ACE index of the experimental group were significantly lower than those of the control group (P < 0.01), and the Shannon index was lower than that of the control group, although the difference was not significant (*P* > 0.05). Simpson’s index was the same as that of the control group. The Chao index and the ACE index of rumen bacteria were decreased in the sheep fed with buckwheat straw and alfalfa fermented by enzymes and bacteria. According to the principal coordinate analysis (PCoA) of the rumen microbial community (Figure 1), PC1 and PC2 contributed 70.44% and 14.45%, respectively, to the variation among samples.

**Table 3.** Number of OTUs and species in each group

Items	Control group	Trial group
Coverage	0.986 ± 0.00^B^	0.988 ± 0.00^A^
observed species	1722.00 ± 86.42	1567.33 ± 146.00
OTUs	1722.00 ± 86.42	1657.33 ± 146.00

The same letter or no letter in shoulder mark of peer data shows no significant difference (*P* > 0.05), different letters show significant difference (*P* < 0.05), and different capital letters show significant difference (*P* < 0.01).The same as blow.

**Table 4.** Alpha diversity detected by 16srDNA

Items	Control group	Trial group
Chao	2787.80 ± 138.34^A^	2457.44 ± 188.74^B^
ACE	2898.43 ± 94.54^A^	2543.55 ± 202.80^B^
Shannon	5.90 ± 0.48	5.81 ± 0.45
Simpson	0.93 ± 0.02	0.93 ± 0.02

The same letter or no letter in shoulder mark of peer data shows no significant difference (*P* > 0.05), different letters show significant difference (*P* < 0.05), and different capital letters show significant difference (*P* < 0.01).The same as blow.

**Figure 2.**
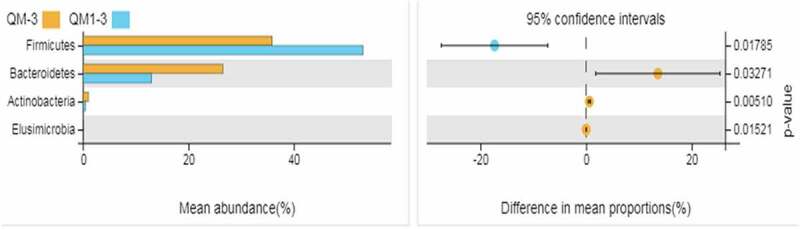
Difference between species via Welch’s t test

Note: QM-3 is the control group and QM1-3 is the experimental group. The same as blow.

Moreover, PCoA analysis showed that the distance between samples in the same group was closer than that between the control group and the experimental group. Table 5 shows that , and bacteroidetes were the dominant bacteria in the control group and the experimental group. The two taxa accounted for more than 90% of the total bacteria. The proportion of Firmicutes in the experimental group was significantly higher than that in the control group (P < 0.05); the proportion of Bacteroidetes was significantly lower than that of the control group (P < 0.01), and the difference in Proteobacteria was not significant (P > 0.05). Figure 2 shows that the bacteria with significant difference between the control group and the experimental group were firmicutes, bacteroides, Actinomycetes, and Elusimicrobia. The proportion of Firmicutes in the experimental group was significantly higher than that in the control group (P < 0.05); the proportions of Bacteroides and Elusimicrobia were significantly lower than that of the control group (P < 0.05), and the number of Actinomycetes was significantly lower than that of the control group (P < 0.01).

**Table 5.** Abundance of dominant bacteria in the whole sample(%)

Phylum	Control group	Trial group
*Proteobacteria*	34.02 ± 2.07	31.72 ± 6.00
*Firmicutes*	35.87 ± 4.01^b^	53.19 ± 0.07^a^
*Bacteroidetes*	26.59 ± 5.21^A^	12.99 ± 5.18^B^

The same letter or no letter in shoulder mark of peer data shows no significant difference (*P* > 0.05), different letters show significant difference (*P* < 0.05), and different capital letters show significant difference (*P* < 0.01).The same as blow.

**Figure 3.**
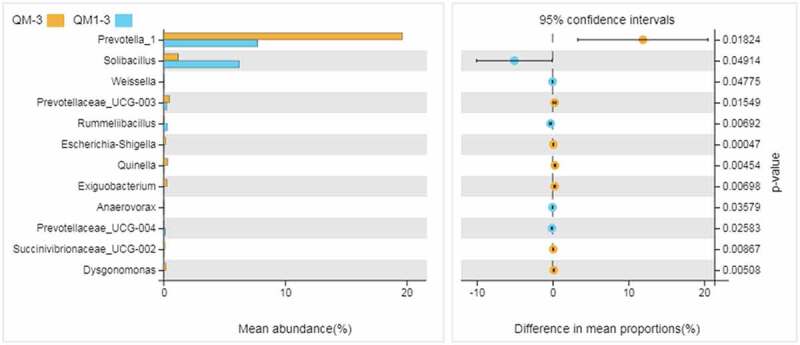
Differences among species via Welch’s t test

Note: QM-3 is the control group and QM1-3 is the experimental group. The same as blow.

The results showed that the numbers of Bacteroides, actinomycetes, and elusimcrobia in rumen bacteria of Tan sheep fed with buckwheat straw and alfalfa fermented by enzyme + bacteria were decreased, while the number of Firmicutes increased. From Table 6, Comamonas, Prevotella_1,Acinetobacter,Lysinibacillus,and Kurthia were the dominant bacteria in the control group and the experimental group. Those taxa accounted for more than 70% of the total bacteria. The proportion of Kurthia in the experimental group was significantly higher than that in the control group (*P* < 0.01). There were no significant differences in *Comamonas, Acinetobacter*, or *Lysinibacillus* between the two groups (*P* > 0.05). Figure 3 shows that there were 12 genera including *Prevotella_1* and *Solibacillus* with significant differences between the control group and the experimental group. The proportions of five genera, Solibacillus, Weissella, *Rummeliibacillus*, *Anaerovorax*, and *Prevotellaceae-ucg-004*, were significantly higher than the corresponding genera in the control group (*P* < 0.05), while the proportions of *Prevotella-1, Prevotellae-ucg-003, Escherichia Shigella, Quinella, Exiguobacterium, Succenivibrionaceae-ucg-002*,and *Dysgonomonas* in the experimental group were significantly lower than those in the control group(*P* < 0.05). The results showed that the amounts of *Prevotella-1, Prevotellaceae-ucg-003, Escherichia Shigella, Quinella, Exiguobacterium, Succenivibrionaceae-ucg-002*, and *Dysgonomonas* were decreased by feeding buckwheat straw and alfalfa after enzyme + bacteria fermentation, and the numbers of *Solibacillus, Weissella, Rummeliibacillus, Anaerovorax and Prevotellaceae-ucg-004* were increased. The 16S rDNA high-throughput sequencing technology uses the sequence diversity in different strains to identify species [23], and thus the method can be widely used for identification without isolation and culturing of microorganisms. This experiment was conducted to study the effects of enzyme + bacteria treatment of buckwheat straw and alfalfa on rumen bacterial flora of Tan sheep. Kong [24] studied the effects of feeding different roughages on the diversity and structure of rumen bacterial flora in dairy cows, and they found that *Firmicutes* and *Bacteroides* accounted for a large proportion of the whole bacterial flora consistent with the results of this experiment. *Firmicutes* is an important group of fiber-degrading bacteria. The proportion of *Firmicutes* increased significantly after the treatment of buckwheat straw and alfalfa by enzyme + bacteria indicating that the digestion and utilization efficiency of roughage in Tan sheep were enhanced after enzyme + bacteria treatment of buckwheat straw and alfalfa.

**Table 6.** Abundance of dominant bacteria in the whole sample(%)

Genus	Control group	Trial group
*Comamonas*	15.44 ± 8.38	20.95 ± 7.72
*Prevotella_1*	19.65 ± 3.81^A^	7.73 ± 3.77^B^
*Acinetobacter*	14.37 ± 7.74	9.24 ± 3.05
*Lysinibacillus*	12.39 ± 1.71	7.24 ± 3.75
*Kurthia*	12.63 ± 3.64^B^	26.04 ± 9.26^A^

The same letter or no letter in shoulder mark of peer data shows no significant difference (*P* > 0.05), different letters show significant difference (*P* < 0.05), and different capital letters show significant difference (*P* < 0.01).The same as blow.

### 3.3 Effects of enzyme + bacteria treatment of buckwheat straw and alfalfa on KEGG metabolic pathways of rumen flora in Tan sheep

**Figure 4.**
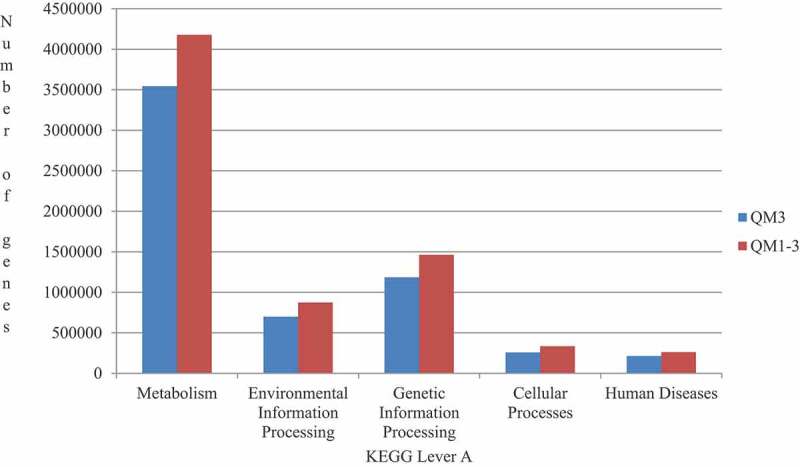
KEGG annotation(level A)

A total of 39,022,896 genes were enriched in 118 KEGG metabolic pathways, and KEGG annotated maps of each sample were obtained by comparison with the KEGG database (Figure 4). Among the A-level genes enriched, the number of genes at the metabolic level was the largest, followed by genetic information processing, environmental information processing, cellular processing and human disease. The results showed that the main functional enzymes in the rumen of Tan sheep fed with buckwheat straw and alfalfa were associated with metabolism, genetic information processing, and environmental information processing. The top 25 most abundant pathways are shown in Table 7. The levels of 15 genes including those associated with purine metabolism, pyrimidine metabolism, two component system, and ABC transporters, in the experimental group were significantly higher than in the control group (*P* < 0.05), while the levels of 7 genes involved in DNA replication, glycolysis/glycolysis and in alanine, aspartate, and glutamate metabolism were not significantly different from the control group (*P* > 0.05), while galactose metabolism and fructose were not significantly different from those in the control group (*P* > 0.05) . Mannose metabolism and phenylalanine, tyrosine, and tryptophan biosynthesis were significantly lower than in the control group (*P* < 0.05). KEGG can systematically study the functions of differentially expressed genes at the molecular level, and thus it is the core of metabolic pathway research [25] . KEGG covers databases containing genome information as well as data concerning disease and signaling pathways; the database integrates various types of information such as data for proteins, genes, and metabolism.

**Table 7.** Ratio of high abundance families of reads to KEGG (level B)

Pathway	Control group	Trial group
Purine metabolism	16302.34 ± 218.68^B^	17140.08 ± 53.88^A^
Pyrimidine metabolism	14464.11 ± 188.29^B^	15473.46 ± 88.33^A^
Two-component system	13005.87 ± 159.65^B^	13411.08 ± 67.78^A^
ABC transporters	11432.27 ± 477.79^B^	13429.50 ± 277.15^A^
Ribosome	11868.33 ± 171.02^B^	12570.48 ± 25.35^A^
Quorum sensing	9730.45 ± 304.23^B^	10476.79 ± 240.40^A^
Starch and sucrose metabolism	10132.83 ± 63.73	10134.92 ± 81.93
Amino sugar and nucleotide sugar metabolism	9924.84 ± 87.16	9913.34 ± 42.73
Homologous recombination	8281.83 ± 73.53	8530.82 ± 34.55
Aminoacyl-tRNA biosynthesis	7869.20 ± 191.02^B^	8739.45 ± 61.82^A^
Glycolysis/gluconeogenesis	7650.30 ± 121.05	7737.06 ± 51.01
Alanine,aspartate and glutamate metabolism	7336.03 ± 76.75	7210.78 ± 86.93
Mismatch repair	6809.54 ± 102.13	6950.78 ± 3.33
Galactose metabolism	6839.14 ± 57.18^A^	6457.29 ± 51.34^B^
DNA replication	6239.30 ± 22.03	6233.71 ± 26.58
Cysteine and methionine metabolism	6484.43 ± 104.75^B^	6702.27 ± 38.39^A^
Peptidoglycan biosynthesis	6101.20 ± 107.23^B^	6479.57 ± 36.86^A^
Pyrimidine metabolism	5676.18 ± 134.75^B^	6095.63 ± 53.77^A^
Glycine,serine, and threonine metabolism	5619.56 ± 136.77^B^	5765.88 ± 60.25^A^
Fructose and mannose metabolism	5471.47 ± 26.79^A^	4947.29 ± 8.74^B^
Oxidative phosphorylation	5110.35 ± 67.54^B^	5187.79 ± 19.71^A^
Phenylalanine,tyrosine and tryptophan biosynthesis	4863.86 ± 31.32^A^	4688.70 ± 72.82^B^
Pentose phosphate pathway	4587.27 ± 119.61^B^	5060.22 ± 3.96^A^
Glyoxylate and dicarboxylate metabolism	4900.22 ± 72.16^B^	5011.26 ± 34.89^A^
Methane metabolism	4112.53 ± 92.70^B^	4611.81 ± 48.42^A^

The same letter or no letter in shoulder mark of peer data shows no significant difference (*P* > 0.05), different letters show significant difference (*P* < 0.05), and different capital letters show significant difference (*P* < 0.01).The same as blow.

By comparison to known pathways. Results concerning differential expression can be classified according to different functions(mainly involving metabolism), as in the present study. Chu Yi [26]studied the changes produced in various metabolic pathways by adding fat and sugar to the basic diet of pigs. The results showed that KEGG enrichment of functional genes was focused on metabolic pathways. Further research and annotation of differentially expressed functional genes involved in those pathways showed that the differences caused by adding fat and sugar were mainly reflected in fatty acid metabolism. In this experiment, the levels of genes involved in purine metabolism, pyrimidine metabolism, ABC transporters, and 15 other genes in the experimental group were significantly higher than those in the control group (*P* < 0.05), indicating that enzyme + bacteria treatment can improve purine metabolism, pyrimidine metabolism, and ABC transporter of Tan sheep. The gene expression of transporters enhanced purine metabolism, pyrimidine metabolism, ABC transporter, ribosome, peptidoglycan biosynthesis, pyruvate metabolism, pentose phosphate pathway, quorum sensing, methane metabolism, and aminoacyl tRNA biosynthesis.

### 3.5 Effects of enzyme + bacteria treatment of buckwheat straw and alfalfa on CAZy enzymes in the rumen of Tan sheep

**Figure 5.**
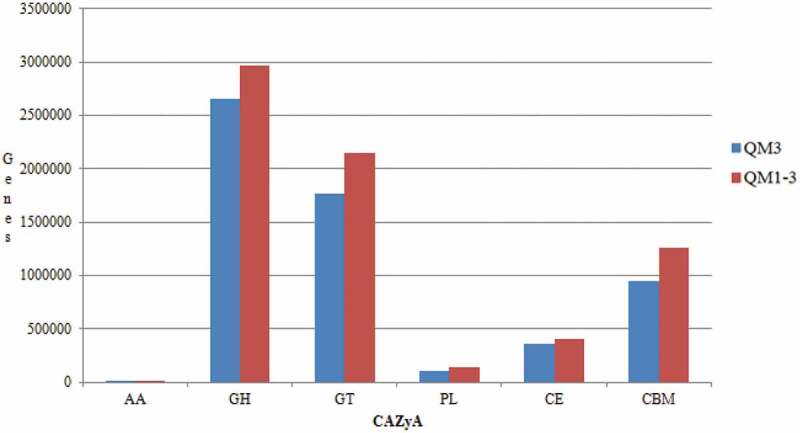
CAZy annotation(level A)

**Figure 6.**
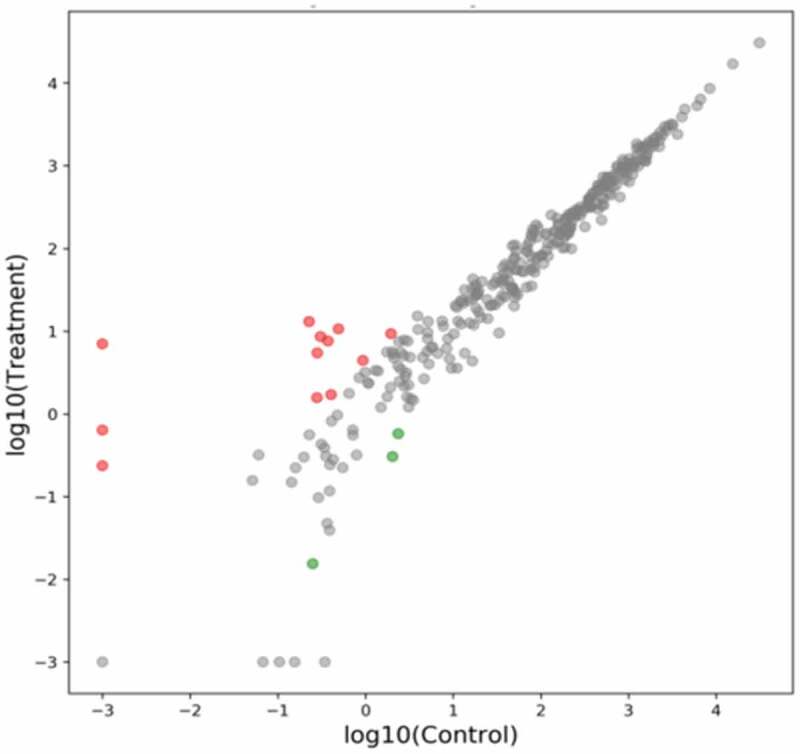
Analysis of CAZy abundance of Deseq2 between the control group and the experimental group

The CAZy database annotation maps for each sample are shown in Figure 5. As can be seen from the figure, the order of the proportion of the experimental group and the control group from high to low was glycoside hydrolase (GHS) > glycosyltransferase (GT) > carbohydrate-binding module (CBMS) > carbohydrate esterifying enzyme (CES) > polysaccharide lyase (PLS) > auxiliary redox enzyme (AAS). The results showed that the main carbohydrate enzymes for degrading plant cellulose in the rumen of Tan sheep fed with buckwheat straw and alfalfa were glycoside hydrolase, glycosyltransferase, and carbohydrate-binding module. For level B, the top five abundances are shown in Table 8. There was no significant difference between the two groups (*P* > 0.05). Based on the analysis of deseq2 abundance, a scatter plot of genes was drawn (Figure 6). In the figure, red indicates significant upregulation of gene expression (*P* < 0.05); green indicates downregulation of gene expression (*P* < 0.05), and gray indicates no change (*P* > 0.05). It can be seen from Figure 6 that the treated buckwheat straw and alfalfa can lead to up or downregulation of gene expression in the rumen of Tan sheep (Table 9). Compared with the control group, the abundances of CBM83 and CBM60 were increased in the experimental group (*P* < 0.05), and the abundances of AA, GH126 and AA12 were downregulated in the experimental group (*P* < 0.05). The Cazy database specializes in the study of carbohydrate-active enzymes (Cazymes). Ruminants can provide energy for the body through the degradation of carbohydrates via enzymes secreted by microorganisms in the rumen. Therefore, researchers have used this database to study the lignocellulose degradation mechanisms of rumen fluid [27]. CAZy enzymes can be divided into six functional groups, among which glucosidase (GH), carbohydrate esterase (CE), and polysaccharide lyase (PL) can synergistically degrade lignocellulose materials [28,29].The present experiment was conducted to study the differences in CAZy enzymes in rumen microbes of Tan sheep after buckwheat straw and alfalfa were treated with enzyme + bacteria. The results showed that the percentage of GH family enzymes in the control group and experimental group was the highest, followed by GT and CBM, while PL and CE accounted for fewer enzymes, consistent with the research results of Hu Dandan [30]. Lynd LR [31] showed that the carbohydrate-binding module (CBM) is an important part of cellulase and that it has a catalytic effect that can promote the combination of enzyme and cellulose, thereby promoting the degradation of cellulose. At present, the CBM contains 84 families. According to the prediction of function from CAZy analysis, CBM6 acts as a cellulose-degrading enzyme [32]. In this experiment, the abundance of CBM6 was high, indicating that Tan sheep can effectively degrade cellulose. The CBM50 family includes genes related to cell wall degradation [33], and this family was found to be related to Bacteroides from the CAZy database. In the present experiment, the abundance of Bacteroides in the experimental group was significantly lower than that in the control group, but there was no significant difference in CBM50 gene level between the experimental group and the control group. The reason may be that a number of other bacterial groups in the rumen of Tan sheep are also related to CBM50. Compared with the control group, some genes in the experimental group were upregulated or downregulated, but these genes were not related to the treatment of buckwheat straw and alfalfa by enzyme + bacteria. However, the specific mechanism needs to be further studied.

**Table 8.** Ratios of high abundance families of reads to CAZy (level B)

LevelA	Lever B	Control group	Trial group
GHs	GH13	234522.33 ± 38501.27	298726.33 ± 44494.17
	GH43	206457.00 ± 33966.33	205827.67 ± 30207.29
	GH35	213555.67 ± 37592.17	160965.67 ± 26533.15
	GH3	158770.33 ± 22828.70	200406.00 ± 29957.74
	GH2	168750.67 ± 26648.80	185595.00 ± 27565.72
GTs	GT2	2161276.00 ± 2500701.36	1056991.33 ± 160265.16
	GT4	1050591.75 ± 1249907.22	548267.67 ± 80354.93
	GT51	256062.00 ± 303796.92	132761.33 ± 19784.53
	GT0	167363.00 ± 194928.96	83135.33 ± 12818.98
	GT68	150532.00 ± 177063.56	76488.67 ± 10768.53
CBMs	CBM50	258514.33 ± 42836.47	318397.67 ± 47961.44
	CBM13	99698.00 ± 16243.93	117958.00 ± 17240.03
	CBM32	81680.33 ± 13184.67	107784.00 ± 17255.26
	CBM6	75331.33 ± 11870.31	92017.00 ± 14040.68
	CBM2	59042.33 ± 9750.56	72814.33 ± 11243.32

The same letter or no letter in shoulder mark of peer data shows no significant difference (*P* > 0.05), different letters show significant difference (*P* < 0.05), and different capital letters show significant difference (*P* < 0.01).The same as blow.

**Table 9.** Differences in CAZy abundance of DEseq2 between groups

Change	Control group vs Trial group
Upregulation	CBM83,CBM60,PL15,GH52,PL24,GH152,CBM53,GT95,CBM8,GT97,GH124,GT72. (*P* < 0.05)
Downregulation	AA1,GH126,AA12. (*P* < 0.05)

## 4. Conclusions

The results showed that the weight gain of Tan sheep could be improved by using buckwheat straw and alfalfa treated by enzymes and bacteria. The dominant bacteria groups in the rumen of Tan sheep were *Proteobacteria, Firmicutes,* and *Bacteroides*. The dominant bacteria were *Comamonas, Prevotella_1, Acinetobacter, Lysinibacillus*, and *Kurthia*. The rumen bacterial diversity of Tan sheep was affected by the treatment of buckwheat straw and alfalfa by enzyme + bacteria fermentation. The main functions of enzymes in the rumen of Tan sheep are metabolism, genetic information processing, and environmental information processing. Glucohydrolase, glycosyltransferase, and carbohydrate-binding module are the main carbohydrate enzymes involved in degradation of plant cellulose. Enzyme + bacteria treatment improved purine metabolism, pyrimidine metabolism, and ABC transporter in Tan sheep. The gene expression of levels of transporters and other genes indicated enhanced purine metabolism, pyrimidine metabolism, ABC transporter, ribosome, peptidoglycan biosynthesis, pyruvate metabolism, pentose phosphate pathway, quorum sensing, methane metabolism, aminoacyl tRNA biosynthesis, and several carbohydrate enzymes in Tan sheep. In this experiment, enzyme + bacteria treatment of roughage was beneficial to the rumen weight gain and the formation of dominant cellulose decomposing bacteria, and the treatment enhanced the expression of some functional genes of Tan sheep.

The results should be beneficial to the healthy breeding of Tan sheep, and the treatment could be widely used in commercial production.

## Highlights

1. The treatment of buckwheat straw and alfalfa by enzyme bacteria is studied.

2. The weight gain of Tan sheep was improved by enzyme and bacteria.

3. The rumen microbial diversity of Tan sheep was changed.

4. Feeding influence the number of some functional genes of rumen microbial flora in Tan sheep.

5. Feeding affected the quantity of carbohydrate enzymes in rumen microbial flora of Tan sheep.

## Research highlights

1. The weight gain of Tan sheep was improved by feeding buckwheat straw and alfalfa treated by enzyme and bacteria.

2. The rumen microbial diversity of Tan sheep was changed by feeding buckwheat straw and alfalfa treated with enzyme and bacteria.

3. Feeding Tan sheep with buckwheat straw and alfalfa treated with enzyme bacteria affected the number of some functional genes of rumen microbial flora in Tan sheep.

4. Feeding Tan sheep with buckwheat straw and alfalfa treated with enzyme bacteria affected the quantity of carbohydrate enzymes in rumen microbial flora of Tan sheep.
